# Control of Gene Expression via the Yeast CWI Pathway

**DOI:** 10.3390/ijms23031791

**Published:** 2022-02-04

**Authors:** Ana Belén Sanz, Raúl García, Mónica Pavón-Vergés, José Manuel Rodríguez-Peña, Javier Arroyo

**Affiliations:** Departamento de Microbiología y Parasitología, Facultad de Farmacia, Universidad Complutense de Madrid, IRYCIS, 28040 Madrid, Spain; absanzsa@ucm.es (A.B.S.); rgarcias@ucm.es (R.G.); monipavo@ucm.es (M.P.-V.); josemanu@ucm.es (J.M.R.-P.)

**Keywords:** transcriptional activation, stress adaptive response, MAPK, gene expression, chromatin, CWI pathway

## Abstract

Living cells exposed to stressful environmental situations can elicit cellular responses that guarantee maximal cell survival. Most of these responses are mediated by mitogen-activated protein kinase (MAPK) cascades, which are highly conserved from yeast to humans. Cell wall damage conditions in the yeast *Saccharomyces cerevisiae* elicit rescue mechanisms mainly associated with reprogramming specific transcriptional responses via the cell wall integrity (CWI) pathway. Regulation of gene expression by this pathway is coordinated by the MAPK Slt2/Mpk1, mainly via Rlm1 and, to a lesser extent, through SBF (Swi4/Swi6) transcription factors. In this review, we summarize the molecular mechanisms controlling gene expression upon cell wall stress and the role of chromatin structure in these processes. Some of these mechanisms are also discussed in the context of other stresses governed by different yeast MAPK pathways. Slt2 regulates both transcriptional initiation and elongation by interacting with chromatin at the promoter and coding regions of CWI-responsive genes but using different mechanisms for Rlm1- and SBF-dependent genes. Since MAPK pathways are very well conserved in eukaryotic cells and are essential for controlling cellular physiology, improving our knowledge regarding how they regulate gene expression could impact the future identification of novel targets for therapeutic intervention.

## 1. Introduction

Yeast cells exposed to environmental stress and diverse molecules can elicit cellular responses that guarantee maximal cell survival. Most of these responses are mediated by signal transduction pathways formed by a mitogen-activated protein kinase (MAPK) module, which are highly conserved from yeast to humans. The MAPK module consists of a cascade of three protein kinases, namely, MAPKKK, MAPKK, and MAPK [1,2]. The final activation of MAPK leads to the phosphorylation of different substrates, including transcription factors, responsible for the rapid transcriptional induction of stress-responsive genes that enable cells to adapt and survive under stress conditions [3].

Five MAPKs mediating stimulus-dependent responses are encoded in the yeast genome (reviewed by [3,4]). Kss1 is required for pseudohyphal and invasive growth upon nutrient starvation, Fus3 regulates mating, Hog1 is necessary to respond to hyperosmotic stress, and Smk1 is involved in sporulation. Slt2/Mpk1 controls cell integrity against cell wall aggressions and other stress conditions that indirectly affect the cell wall, including heat shock, hypo-osmotic shock, hyper-osmotic shock, high and low pH, arsenate, cadmium, or plasma membrane, ER (endoplasmic reticulum), oxidative, and genotoxic stresses [5,6]. Kss1 also mediates cell integrity in response to defects in protein glycosylation. The specific response of each MAPK pathway is mainly regulated by the presence of particular receptors and osmosensors on the cell surface. These guide the stress signal towards the appropriate MAPK module and the corresponding transcription factors, triggering stimulus-dependent responses. Tight regulation of signaling circuits is also essential to guarantee an adequate gene expression and avoid an alteration in cell physiology [7,8]. An essential way of regulating these pathways is at the level of MAPK dephosphorylation by protein phosphatases [9,10]. However, other negative and positive feedback mechanisms attenuate or exacerbate these responses [3,11,12,13]. 

The activation of rapid and efficient transcriptional adaptive responses depends not only on MAPKs and transcription factors, but also on protein complexes that modulate chromatin structure. Transcriptional responses triggered by different stresses share certain regulatory features, but others are unique to each type of stress. In this paper, we review the main molecular mechanisms that regulate gene expression in response to cell wall stress as an example of a regulated transcriptional response mediated by a conserved MAPK pathway, considering the naturally repressive state of chromatin.

## 2. Chromatin and Gene Expression in Response to Stress

The regulation of gene transcription in eukaryotic cells involves a dynamic balance between the packaging of regulatory sequences into chromatin and the access of transcriptional regulators to these sequences [14,15]. Chromatin, the native compacted form of DNA, is organized into various condensation levels. The first level of compaction is the nucleosome, consisting of a 147 pair base DNA fragment wrapped around an octamer of the four core histones H2A, H2B, H3, and H4, establishing 14 contacts between the positively charged residues in the histones and the phosphate backbone of the DNA [16,17,18]. Nucleosomes are then linked together by a variable length of linker DNA associated with H1 linker histone. These structures can be compacted into higher-order structures like the 30 nm fibers that are transcriptionally inactive. This can be successively folded until reaching its maximum degree of compaction in metaphase chromosomes. Each histone has a globular domain and an amino (N)-terminal tail of between 16 to 44 residues in length. Histone tail regions are not critical for nucleosome integrity [19,20], however, they can protrude from their nucleosome and interact with other nucleosomes, playing a significant role in chromatin compaction (for review, see [21,22]).

To overcome the naturally repressive state of the chromatin structure and promote transcriptional activation, nucleosome removal at the promoters of stress-responsive genes is a critical step. Nucleosome positioning can influence the accessibility of DNA binding sites in the promoters to specific transcription factors [23,24,25] and interfere with the assembly and progression of the general transcriptional machinery, acting as an extra level of regulation [26,27,28,29,30,31]. For example, most of the transcription factors involved in the heat stress response (Hsf1, Msn2, Msn4, and Aft2) showed significant increases in their accessibility because of nucleosome repositioning upon heat shock [32,33]. In this context, post-translational modifications of individual histones by histone modifiers (reviewed in [34,35]) and disassembly and removal of nucleosomes by ATP-dependent chromatin remodeling complexes [36] work in concert to regulate this process by rendering promoters accessible to Pol II [37,38,39]. Moreover, histone modifications can stimulate the recruitment of chromatin-modifying enzymes at specific genomic sites, allowing an increase or decrease in other histone modifications that can propagate to adjacent nucleosomes by several positive and negative feedbacks [40]. Thus, chromatin is a critically important component of the cellular stress responses, mediating their speed and amplitude [41,42]. 

Chromatin modulation via covalent histone modification, such as methylation, acetylation, phosphorylation, SUMOylation, ADP-ribosylation, among others, represents one fundamental way to regulate DNA accessibility during gene transcription and many other cellular processes [22,43,44,45,46,47]. These modifications regulate chromatin structure and provide a signaling platform to recruit downstream effector proteins belonging to the transcriptional machinery and chromatin remodeling complexes [43,48]. 

Histone acetylation is regulated by the opposing action of histone acetyltransferases (HATs) and histone deacetylases (HDACs). SAGA (Spt-Ada-Gcn5-acetyltransferase) is a highly conserved co-activator complex that controls transcription by modifying histones [49]. It is a large multisubunit complex (19 subunits) organized into four functionally distinct modules: the HAT module (the catalytic subunit Gcn5, Ada2, Ada3, and Sgf29), the deubiquitination (DUB) module (the ubiquitin-specific protease Ubp8, Sgf11, Sgf73, and Sus1), the core structural module (including Taf and Spt proteins), and the transcription factor-binding module (Tra1) [50,51]. Acetylation by Gcn5 utilizes acetyl CoA as a cofactor and catalyzes the transfer of an acetyl group primarily to the K14 and K9 H3 residues [52]. This modification has long been positively correlated with an open chromatin conformation and gene expression under stress conditions [53,54]. Indeed, Gcn5 is generally recruited to the promoter of active genes [55]. Acetylation of histones neutralizes the positive charge of the histone tails. It decreases their affinity for negatively charged DNA, affecting higher-order chromatin packing that would otherwise be inhibitory for regulatory factor recruitment and transcription. This modification also creates specific binding surfaces for bromodomain-containing proteins, proving that acetyl-lysines in histones recruit or stabilize the machinery involved in DNA-mediated processes [56,57,58]. However, other SAGA subunits, such as Spt3 and Spt8, enhance pre-initiation complex assembly by delivering TBP (TATA-binding protein) to promoters, a mechanism that goes beyond HAT activity [59,60,61] and that has been related to the expression of highly regulated stress responsive-genes [62,63]. Finally, within the deubiquitination DUB module, Ubp8 catalyzes the deubiquitination of histone H2B, an important step for gene activation [64,65].

Besides histone modifiers, ATP-dependent chromatin remodeling complexes are needed to disrupt DNA-histone interactions using the energy from ATP hydrolysis [66]. The remodeler action can result in nucleosome sliding (octamer position varies across the DNA), nucleosome eviction (ejection), or localized nucleosome unwrapping (remodeled state in which DNA is more accessible but histones remain attached). Additionally, they can also alter nucleosome composition by dimer histone replacement [36]. 

There are four different families of chromatin remodeling complexes: SWI/SNF, INO80/SWR1, ISWI, and CHD. They share a similar ATPase subunit that belongs to the SF2 superfamily of ATPases characterized by an ATPase domain split into two parts, the DExx and HELICc regions [67], although they are specialized for particular purposes. The yeast SWI/SNF family, including SWI/SNF and RSC, are large chromatin remodeling machines that can move or eject nucleosomes, facilitating transcription and other nuclear processes [68]. *S. cerevisiae* SWI/SNF consists of 11 subunits, with Snf2 serving as the ATPase subunit [69,70]. Targeted recruitment of the SWI/SNF complex can be achieved via direct interaction with gene-specific activators [71,72,73,74] to locally alter nucleosome positioning at the promoter, facilitating the binding of transcription factors to DNA, and then stimulating transcriptional initiation by Pol II [72,73].

Transcription initiation is a complicated process that requires the coordinated activities of co-activators and many factors to ensure appropriate gene regulation under stressful situations [38,75]. Chromatin remodeling and histone modification have proven to be key players in transcriptional regulation. They are not mutually exclusive as their activities could be complementary to support stress-responsive gene induction. Over the past few years, the involvement of different chromatin-modifying and remodeling activities in response to osmotic stress [76,77,78,79,80], heat stress [32], diamide [41], ethanol [81], and cell wall stress [82,83] have been characterized. Particular post-translational modifications required for transcriptional stress responses have been identified. For instance, H3K4 monomethylation dictates nucleosome dynamics and chromatin remodeling of stress-responsive genes [84]. Some histone modification patterns under various stress conditions have also been defined by high-throughput phenotype analysis of histone residues upon DNA damage and heat stress [85], phosphate starvation [86], or diamide treatment [41,87]. In a recent study, a global map of the histone residues required for transcriptional reprogramming in response to heat and osmotic stress was elucidated [88]. Together, these studies suggest a personalized, rather than general, subset of histone requirements for each chromatin context.

MAPKs mainly control gene expression by phosphorylating transcription factors. This modification regulates its activity in different ways, including their protein levels, binding to DNA, translocation between nucleus and cytoplasm, and interaction with regulatory proteins [89]. However, the role of MAPKs in regulating these responses is not only restricted to the phosphorylation of the transcription factors. MAPKs may also associate with chromatin to recruit different regulatory elements and be integral components of transcriptional activation complexes on gene promoters [90]. In fact, various studies have shown that MAPKs in yeast and mammals are recruited to gene promoters and coding regions in response to stress. In yeast, active Hog1 associates with osmostress-responsive promoters by direct interaction with transcription factors to mediate the recruitment of general transcription factors, chromatin-modifying activities, and RNA Pol II during transcriptional initiation. However, it also associates with coding regions, having an important role in elongation (see [91] for review). p38, the homolog of Hog1 in mammals, is also recruited to both the promoter and open reading frames (ORFs) of target genes in response to anisomycin, an inhibitor of protein synthesis [92].

Genome-wide analyses demonstrated that, in addition to Hog1, Fus3, and Kss1, other kinases of the mating pheromone signaling pathway are physically associated with coding regions of pheromone responsive genes. Strikingly, upstream MAPK proteins can also associate with chromatin regions [92,93,94]. In yeast, Ste5, the central scaffold protein of the pheromone response pathway, occupies the same genes as Fus3 and Kss1 upon pheromone stimulation, suggesting that adaptor proteins might also be involved in protein interactions of nuclear localization [94,95]. Moreover, stress-activated MAPK Sty1 is recruited to promoters by the Atf1–Pcr1 transcription factor complex to regulate stress-induced gene expression in the fission yeast *Schizosaccharomyces pombe* [96,97]. 

Initially, genomic approaches did not find evidence for Slt2 gene occupancy under some specific conditions of Slt2 activation (cell cycle and pheromone exposure) [94]. However, later on, it was shown that, in response to cell wall stress, this MAPK interacts with chromatin at the promoter and coding regions of CWI-responsive genes regulated both by SBF (Swi4/Swi6) [98,99,100] and Rlm1 transcription factors [101]. Consequently, Slt2 participates in two well-differentiated mechanisms, discussed below, to regulate both transcriptional initiation and elongation.

## 3. The CWI Pathway and Regulation of Gene Expression

**Figure 1:**
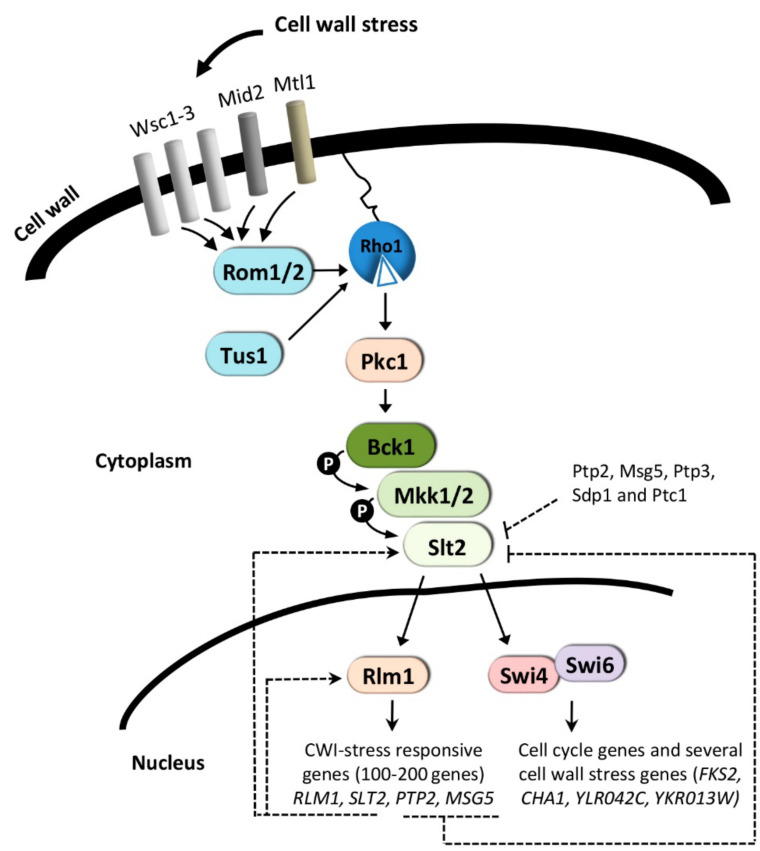
Cell Wall Integrity (CWI) pathway. Cell wall damage is sensed at the plasma membrane via cell-surface proteins that stimulate nucleotide exchange on Rho1 and activation of Pkc1. The main role of activated Pkc1 is to trigger the MAPK module (Bck1, Mkk1/Mkk2, and Slt2). Phosphorylation of Slt2 leads to the activation of the transcription factors SBF (Swi4/Swi6) and Rlm1. SBF is mainly involved in regulating genes during G1/S transition, whereas Rlm1 is responsible for the transcriptional activation of most of those genes induced in response to cell wall stress. Rlm1 elicits transcriptional positive feedback loops on the expression of *RLM1* and *SLT2*, which result in the amplification of gene expression levels of CWI-responsive genes. In contrast, the Rlm1-dependent transcriptional induction of the Slt2 phosphatases, Ptp2 and Msg5, attenuates the induction of the CWI pathway, negatively modulating the CWI transcriptional activation response. In addition, other Slt2 phosphatases, like Ptp3, Sdp1, and Ptc1, also contribute to this attenuation. Arrows and T symbols represent activation (positive) and inhibitory (negative) events, respectively.

The yeast cell wall is an essential structure surrounding the cell, necessary for maintaining cell morphology and viability. Stressful conditions damaging this structure trigger the CWI pathway coordinated by the MAPK Slt2/Mpk1 (reviewed in [5]) (Figure 1). As its name implies, the CWI pathway of the budding yeast *Saccharomyces cerevisiae* has a main role in the regulation of cellular responses to cell wall damage and has been well characterized with regard to its regulation by cell wall stress [5,102,103]. Additionally, this pathway is important to regulate morphogenetic events that involve cell wall remodeling and actin cytoskeleton organization during cell cycle progression. The mechanisms by which the CWI pathway regulates the main cell-cycle transitions in response to cell-surface perturbance to delay cell-cycle progression have been recently updated in an excellent review by Quilis and co-workers [104]. Slt2 also participates in the regulation of iron homeostasis. Slt2 phosphorylates and negatively regulates Aft1 activity upon iron deprived conditions, suggesting that the MAP kinase is involved in the regulation of Aft1 activity in a feedback mechanism destined to modulate gene expression in response to iron availability [105].

### 3.1. Cell Wall-Stress Conditions

Under cell wall stress conditions induced by treatments with cell wall perturbing agents that interfere with the biogenesis of this structure, Mid2 and Wsc1, the primary sensors of this pathway, interact with the guanine nucleotide exchange factor Rom2, activating the small GTPase Rho1, which then interacts with and activates Pkc1. The central role of activated Pkc1 is to trigger a MAPK module comprising MAPKKK Bck1, the redundant MAPKKs Mkk1/Mkk2, and the MAPK Slt2. Phosphorylation of the MAPK Slt2 leads to activation of the transcription factors SBF (Swi4/Swi6) [106] and Rlm1 [107] (Figure 1). SBF is primarily involved in gene regulation during G1/S transition, but it also drives the gene expression of a minor group of genes in response to cell wall stress in a manner that is independent of its role in G1-specific transcription [98,100] (discussed below). Rlm1 is responsible for the induction of the primary CWI-adaptive transcriptional response, which has been extensively studied by genome-wide expression profiling (reviewed in [102]). These studies allowed the characterization of the transcriptional programs to cell wall-perturbing agents that interfere with cell wall integrity by different mechanisms, including Congo Red [108], Calcofluor White [109], or poacic Acid [110] which binds to cell wall polymers, inhibiting cell wall construction, zymolyase [111] which alters the cell wall via its β-1,3-glucanase, protease, and chitinase activities, or echinocandins which inhibit β-1,3-glucan synthase [112,113,114]. Changes in the transcriptional profiles of 100-200 genes include a CWI transcriptional fingerprint. This signature comprises the induction of genes related to cell wall biogenesis and remodeling, metabolism and energy, morphogenesis, signal transduction, and stress, required to compensate cell wall defects [102,115]. The final consequence of this response, known as “compensatory salvage response”, is to provide the cell with the mechanisms for the synthesis and crosslinking of cell wall polymers necessary for the maintenance of cellular integrity and fungal survival [5,102]. 

Although the CWI pathway plays a crucial role in regulating these responses, other pathways are also necessary to overcome cell wall stress situations. The transcriptional response elicited by Congo Red depends almost entirely on the MAPK Slt2 and the transcription factor Rlm1 [83,108]. Most of the adaptive transcriptional response to zymolyase also involves Rlm1, but sequential activation of the HOG and CWI pathways is required in this case [111,116]. Glycerol seems to be a key mediator of the crosstalk between CWI and HOG pathways. Activation of Slt2 in response to zymolyase treatment is a consequence of Hog1-driven glycerol accumulation [117]. Moreover, heat-shock activates Hog1 via the CWI pathway, and the main role of the CWI in this process is to stimulate glycerol loss [118]. Ssk2 is also necessary for full Slt2 activation in response to SDS, supporting the necessity of an interplay between HOG and CWI pathways to cope with this stress [6]. Crosstalk between the CWI and cAMP-PKA signaling pathways controls the *TPK1* expression in response to heat stress. The CWI pathway may be activated starting from Mkk1 of the MAPK cascade, resulting in an appropriate PKA output. Wsc3 would be the sensor of this lateral input in a way that seems independent of the activation of upstream elements of the CWI route [119].

The plant natural product poacic acid specifically binds to β-1,3-glucan and triggers a transcriptional response co-regulated by parallel activation of the CWI and HOG signaling pathways [110]. Alternatively, inhibition of β-1,3-glucan synthesis by echinocandins induces the activation of a CWI transcriptional response dependent on both Slt2 and Rlm1, and a parallel response independent of both elements elicited by the inhibition of PKA signaling [120]. 

The MAPK Slt2 activates Rlm1 by phosphorylation, which is required for a proper CWI transcriptional response. However, *SLT2* and *RLM1*-mediated positive feedback mechanisms are also needed for a complete transcriptional activation (Figure 1). Both genes are induced by cell wall stress in an Rlm1-dependent manner [83,108,121]. Thus, Rlm1 elicits a positive transcriptional feedback mechanism enhancing its production rate, amplifying and slowing down gene expression kinetics. Abrogation of the autoregulatory feedback mechanism exerted on *RLM1* severely affects the transcriptional response elicited by CWI pathway activation. In contrast, the blockade of the positive feedback mechanism on *SLT2* affects overexpression of *SLT2* but not *RLM1,* having less impact on the CWI output response [122]. Therefore, phosphorylation of Rlm1 by Slt2 is critical but not sufficient for a complete functional CWI transcriptional response, which requires concurrent *SLT2* and *RLM1*-mediated positive feedback mechanisms. Additionally, negative feedback events contribute to attenuate CWI responses, including the transcriptional induction of *PTP2* and *MSG5* by cell wall stress in an Slt2-dependent manner (Figure 1).

#### 3.1.1. Transcriptional Activation Mechanism for SBF-Dependent Genes

**Figure 2:**
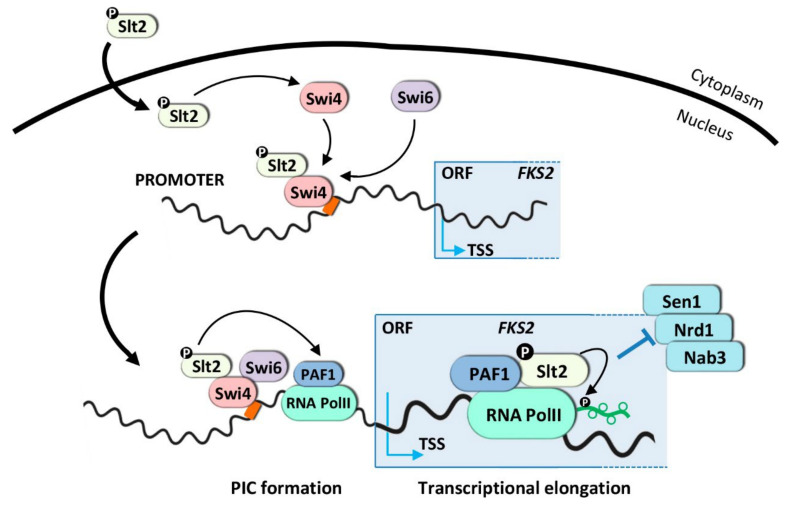
Transcriptional activation mechanism for SBF-dependent genes. Under inducing conditions, Slt2 and its pseudokinase, Mlp1 (not shown), are phosphorylated by Mkk1/2. Slt2 uses a non-catalytic mechanism to activate transcription of SBF-dependent stress-induced genes by recruitment of initiation factors to target promoters. Once activated, Slt2 and Mlp1 interact with Swi4 to bind to the *FKS2* promoter. Then, Swi6 is recruited to form a complex that permits the assembly of RNA Pol II and Paf1C elongation complex. This mechanism requires the activation of Slt2 but not its catalytic activity. Slt2 also serves a function in transcription elongation, moving from the initiation complex (PIC) to the elongation complex on the Paf1C scaffold. Slt2 association with Paf1 overcome transcriptional attenuation by blocking recruitment of the Sen1-Nrd1-Nab3 termination complex. Additionally, phosphorylation of the Tyr1 residue at the RNA Pol II CTD by Slt2 could also be involved in controlling Nrd1/NNS function.

SBF (Swi4/Swi6) is one of the transcription factors involved in CWI signaling. Swi4 is the sequence-specific DNA binding subunit, whereas Swi6 is the transcriptional activation subunit. However, Swi6 is necessary for the binding of Swi4 to DNA by relieving an auto-inhibitory intramolecular association of the Swi4 C-terminal domain with its own DNA binding domain [123,124]. SBF principally regulates G1-specific transcription genes [125,126]; however, it is also involved in the expression of a small subset of CWI genes under elevated growth temperature, including *FKS2*, *CHA1*, *YLR042C*, and *YKR013W.* This induction is mediated via a non-catalytic mechanism proposed by Levin and colleagues [98,99,100,127] (Figure 2). Upon heat shock, Slt2 binds to the promoter and ORF of *FKS2* independently of its protein kinase activity through association with Swi4, but dependent on its activation by upstream activating kinases. This mechanism requires the phosphorylation of the MAPK Slt2 and/or its pseudokinase Mlp1 to interact with Swi4 and form a complex that associates with SBF-binding sites in the promoters of CWI-dependent genes, independently of Swi6. In this process, the catalytic activity of Slt2 and, therefore, the phosphorylation of the transcription factor Swi4 is not required. Although Slt2 relieves the autoinhibitory Swi4 interaction, Swi6 needs to be directed by this complex to the DNA for the recruitment of RNA Pol II (Figure 2). Direct regulation of Swi4 by Slt2 through this non-catalytic mechanism is independent of the role of SBF in cell cycle-regulated transcription [104].

In addition to their role in transcription initiation, Slt2 and Mlp1 also participate in elongation, traveling along the coding region associated with Pol II and Paf1C. In the model proposed, Slt2 and Mlp1 move from the transcription initiation complex to the transcription elongation complex, leaving Swi4-Swi6 behind [99]. Slt2 associates with the Paf1 complex at the *FKS2* promoter. This interaction does not require the catalytic activity of the MAPK, however, it does require the presence of Swi4/Swi6 to direct this association with a restricted group of genes regulated by Slt2 and SBF. Slt2 interacts with the Paf1 complex via a docking site motif in the Paf1 subunit, but its catalytic activity is dispensable for this binding. In consequence, mutations in this domain block the transcriptional elongation of *FKS2*. The recruitment of Pol II (Rpb3) to the *FKS2* promoter upon heat stress is not impaired in a *paf1-4A* mutant; however, the polymerase does not progress to the coding region, indicating a transcription elongation defect. The association between Slt2 and Paf1 blocks the recruitment of the Sen1-Nrd1-Nab3 termination complex and, therefore, avoids premature termination of the *FKS2* gene [99] (Figure 2). All these results indicated that Slt2, for the first time, was part of the transcription initiation and elongation machinery bound to DNA. Interestingly, human ERK5 complements the loss of Slt2, mediating this non-catalytic transcriptional mechanism. Moreover, human Paf1 complements the Slt2-dependent function of yeast Paf1, suggesting that ERK5, and perhaps other MAPKs, possess non-catalytic tasks that require a signal from upstream kinases. Moreover, the regulatory mechanism where Slt2 overcomes transcriptional attenuation by blocking the Sen1-Nrd1-Nab3 termination complex to the elongating polymerase seems to be conserved in humans [98,99].

In contrast to a non-catalytic role for Slt2 in *FKS2* transcription elongation, Yurko and co-workers demonstrated that Slt2 phosphorylates the Tyr1 residue of the C-terminal domain (CTD) of RNA Pol II, increasing Tyr1P levels in response to stress [128]. These effects are accompanied by defects in transcription termination factor recruitment. These authors showed that Nrd1 occupancy relative to Rpb1 at the *FKS2* promoter is reduced following heat shock in wild-type cells, but increases when the Tyr1 residue changes to Phe, indicating that phosphorylation of Tyr1 by Slt2 is required for Sen1-Nrd1-Nab3 (NNS) complex loss upon heat shock (Figure 2). Therefore, Tyr1 phosphorylation impairs Nrd1 recruitment to chromatin, suggesting that it is the phosphorylation of Tyr1 by Slt2 associated with Paf1C which impairs termination factor recruitment to RNA Pol II [128,129]. 

#### 3.1.2. Transcriptional Activation Mechanism for Rlm1-Dependent Genes

**Figure 3:**
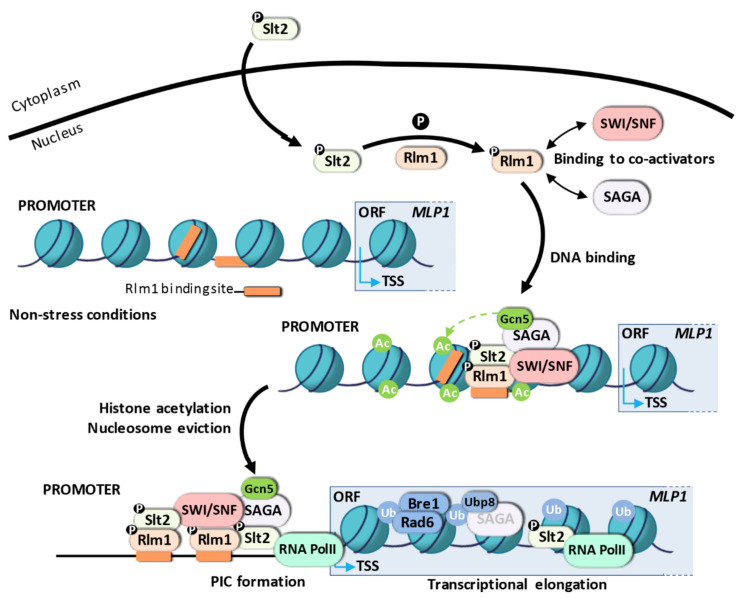
Transcriptional activation mechanism for Rlm1-dependent genes. Under cell wall stress conditions, activated Slt2 phosphorylates Rlm1. Rlm1 interacts with SWI/SNF and probably the SAGA complexes to direct both to the promoters of CWI responsive genes. Upon recruitment, SAGA acetylates histone H3 and cooperates with SWI/SNF to locally alter nucleosome positioning at the *MLP1* promoter, facilitating the binding of Rlm1 to its binding sites, previously occluded by positioned nucleosomes, in a two-step mechanism. Slt2, also attached to the DNA, interacts with RNA Pol II traveling along the coding region during transcriptional elongation. During elongation, ubiquitination of H2B by Rad6 at the coding region is necessary for H2B displacement in response to stress. The ubiquitin protease Ubp8 regulates deubiquitination of H2B and is recruited to the coding regions of CWI-responsive genes for maintaining appropriate levels of H2B ubiquitination.

Rlm1 is the transcription factor responsible for the expression of most of the genes (~90%) induced under cell wall stress [83,108,130]. The *RLM1* (Resistant to the Lethality of constitutive Mkk1) gene was first identified in a genetic screening for mutants resistant to the growth inhibition caused by a constitutive form of Mkk1 [131]. Rlm1 is a MADS (Mcm1-Arg80-Deficiens-serum response factor) box transcription factor related to a member of the mammalian MEF2 family of transcriptional regulators, sharing the same DNA-binding specificity in vitro (TAWWWWWTAGM, W as thiamine or adenine and M as adenine or cytosine; [109,132]). Rlm1 is phosphorylated in vitro by Slt2 and in vivo under heat shock in an Slt2-dependent manner [107]. Mutation of three potential phospho-acceptor sites S374, S427, and T439 inside Ser/Thr-Pro motifs of the transcriptional activation domain of Rlm1 abrogates gene expression of a CWI related gene in response to Calcofluor White [133]. Rlm1 is always located at the nucleus of yeast cells [133], so its activation by the MAPK Slt2 must take place here. A combination of gene expression analysis, chromatin immunoprecipitation (ChIP), and nucleosome scanning assays allowed us to describe a model for the sequence of events during transcriptional activation upon cell wall stress, where the packaging of regulatory sequences into chromatin plays an essential role [82,83,101] (Figure 3). 

To understand the transcriptional activation mechanism regulated by the CWI pathway, it is essential to know the structure of gene promoter targets. *MLP1/KDX1* is one of the genes induced under all cell wall stress conditions tested so far [102,115]. This gene is a model of CWI reporter genes because it shows low basal gene expression levels, but is highly expressed under cell wall stress, and this induction is largely dependent on Slt2 and Rlm1 [108]. The *MLP1* promoter has two functional binding sites for Rlm1, BOX1 (-359/-350) and BOX2 (-510/-501). The nucleosome pattern characterized for the *MLP1* gene revealed the presence of four nucleosomes positioned within the −699 and +161 region under non-stress conditions, the BOX1 site being exposed in the linker DNA between nucleosomes -3 and -2, or partially exposed at the edge of nucleosome-2, whereas the BOX2 site is completely occluded by nucleosome -3, preventing, or at least hindering, the binding of Rlm1 to this sequence [83]. Genome-wide analysis also reveals the existence of both occluded and exposed binding sites for Rlm1 in other CWI genes [134]. The structure of the *MLP1* promoter corresponds to that observed in covered promoters or nucleosome-occupied promoters, identified in most stress-induced genes (reviewed in [14]).

The pattern of promoter nucleosome occupancy is correlated with the capacity of genes to alter their expression. In contrast to housekeeping genes, the promoters of genes that modify their expression levels in response to external and internal signals present nucleosomes covering the transcription start site (TSS), the regions adjacent to the TSS, and most of the binding sites for transcriptional activators, being highly regulated by different remodeling complexes and/or chromatin modifiers [26,135,136]. The *PHO5* gene has a covered promoter and is one of the first models established for gene regulation via chromatin remodeling complexes [137]. The covered promoters are also characterized by presenting a TATA box, present mainly in stress-responsive genes, rather than in housekeeping genes. However, as an exception to most stress-regulated genes that carry a closed promoter, the *MLP1* gene lacks a TATA box [138], although the binding of TBP to the *MLP1* promoter would be jointly regulated by TFIID and SAGA complex [62]. 

Under cell wall stress conditions, the activated MAPK Slt2 phosphorylates Rlm1, resulting in its binding to specific sequences within target promoters (Figure 3). In fact, a yeast strain expressing a version of Rlm1 mutated at the ten potential phosphorylation sites for MAPKs or a catalytically inactive Slt2 results in a lack of Rlm1 enrichment at CWI genes and, consequently, gene induction [83,101]. Therefore, the role of Slt2 is essential in the phosphorylation of Rlm1 to trigger the transcriptional reprogramming via a catalytic mechanism different from that observed for SBF-dependent genes. Furthermore, the best-understood mechanism by which other MAPKs modulate transcription initiation is the phosphorylation of specific transcription factors. In response to osmostress, Hog1 regulates gene expression through different activators (Hot1, Smp1, Msn1, Msn2/4) and the Sko1 repressor that can act independently or in combination at specific promoters via different mechanisms [139]. Smp1 and Sko1 interact with Hog1 and are directly phosphorylated by Hog1. This phosphorylation turns Sko1 into an activator, modifying its association with the co-repressor Tup1-Ssn6 and allowing the recruitment of SAGA and SWI/SNF complexes to target genes [140,141]. On the other hand, Hog1 interacts with Msn2/4 and Hot1, allowing the attachment of the MAPK to promoters dependent on its catalytic activity, although gene expression is non-dependent on transcription factor phosphorylation [142,143], indicating that the catalytic activity of Hog1 per se is a crucial requirement for Pol II recruitment and, therefore, for transcription initiation. 

The assembly of a pre-initiation complex at CWI-responsive genes must require chromatin-modifying activities to remodel or evict nucleosomes positioned along the target promoters (Figure 3). A connection between SWI/SNF, SAGA, and the cell wall stress-triggered transcriptional response was first established in a large-scale screening using the entire collection of haploid deletion strains transformed with a CWI reporter system (p*MLP1*-*NAT1*) [83]. Indeed, 76% of genes induced under cell wall stress are dependent on the SWI/SNF complex [83]. The ATP-dependent chromatin remodeling complex SWI/SNF is essential to displace nucleosomes positioned at the occluded Rlm1-binding sites and surrounding regions to permit Rlm1 entry and Pol II assembly. In the *MLP1* gene, the exposed Rlm1 binding site would allow Rlm1 access to the promoter in a first step, but nucleosome displacement would be required upon stress to expose the additional occluded site in a two-step model for activation [83] (Figure 3). The physical interaction between Rlm1 and SWI/SNF and the requirement of Rlm1 for SWI/SNF recruitment indicates that the remodeler is directed to the promoters via interaction with the transcription factor Rlm1 [83]. It has been reported as the general mechanism for the recruitment of the SWI/SNF remodeling complex to target gene promoters since they cannot recognize specific DNA sequences by themselves [71,72,73]. This interaction occurs at the nucleus, and together they bind the exposed site to locally alter chromatin, ejecting the four nucleosomes positioned at the *MLP1* promoter [83].

Cooperation between SAGA and SWI/SNF for an efficient transcriptional response under cell wall stress has been well established. Genome-wide expression analysis reveals a co-regulation of 65% of induced genes [82]. Indeed, the SAGA complex is recruited to the promoter of these genes in an Slt2 and Rlm1-dependent manner, probably through Rlm1 as deduced by the interaction between either Gcn5 or Ada2 (two SAGA subunits) and Slt2 in an Rlm1-dependent manner [99] (Figure 3). SAGA binding also depends on SWI/SNF but not vice versa, so it seems that SWI/SNF recruitment is necessary for the entry of SAGA. However, simultaneous entry of both complexes cannot be ruled out because no temporal differences in binding have been described [82]. In these promoters, SAGA acetylates the H3 histone, the HAT activity of Gcn5 being essential for *MLP1* gene expression upon cell wall stress (Figure 3).

Although H3 acetylation by SAGA is not critical for pre-initiation complex assembly (Pol II, Snf2, and Rlm1 binding are only slightly affected in SAGA mutants), it increases the remodeling mediated by the SWI/SNF complex. Indeed, the simultaneous deletion of *SNF2* and *GCN5*, in addition to blocking chromatin remodeling, Rlm1 binding, and gene expression, also increases hypersensitivity to cell wall stress compared with individual deletions [82]. As already mentioned, histone acetylation can also create a target for proteins containing bromodomains, including Gcn5 and Spt7 of the SAGA complex, and Snf2 of the SWI/SNF complex. Thus, besides its role in nucleosome unpacking, this mark will stabilize SAGA itself, although it is unlikely to stabilize the SWI/SNF complex because the recruitment of Snf2 is almost unaffected in the absence of Gcn5 upon cell wall stress. The SAGA complex is also recruited to osmostress genes, playing a selective role under severe osmostress conditions [79]. However, histone deacetylation mediated by the Rpd3 HDAC enzyme is required in this case, and in response to oxidative and heat stress, to induce gene expression [76,144,145]. This points to a different chromatin-regulated process in which a decrease in histone acetylation could provide unique binding motifs for the recruitment of other activators to promote gene expression.

Parallel recruitments of different co-activators have been observed in the transcriptional initiation of stress-inducible genes, probably to support the rapid and fine-tuned upregulation required during adverse conditions. For example, simultaneous recruitment of SAGA and SWI/SNF has also been observed at osmotic stress-inducible genes [140] and the glucose-repressed *SUC1* gene [146], and even different ATP-dependent chromatin remodeling complexes such as SWI/SNF, RSC, and ISWI work together to regulate the expression of heat shock genes [147]. The RSC remodeling complex has also been related to cell wall stress, although its precise role in gene expression has not been elucidated [148,149,150]. Together with histone acetylation, histone methylation and protein ubiquitination have been proven important to regulate gene expression via the HOG pathway. Monomethylation of the H3K4 histone by the histone methyltransferase Set1 dictates the specificity of chromatin remodeling, acting the RSC complex in the presence of monomethylated H3K4 and the SWR-C chromatin remodeling complex in the absence of H3K4 monomethylation [84]. Moreover, Ubp3, a ubiquitin-specific protease, is recruited to the promoters and coding regions of osmostress responsive genes to mediate protein ubiquitination in a Hog1-dependent manner, indicating that the balance of ubiquitinated proteins is important for its transcriptional initiation and elongation [80]. 

In response to cell wall stress, Slt2 binds to promoters and to the coding regions of CWI genes [101]. The association of Slt2 with DNA relies on its interaction with Rlm1 and requires an active Slt2 MAPK and the participation of chromatin-modifying complexes. Slt2 could be attached to the chromatin to mediate the phosphorylation of Rlm1, or it could also drive additional mechanisms in transcription initiation or elongation. Unlike the other MAPKs, Slt2 and its human ortholog ERK5 present a transcriptional activation domain within the C-terminal region [151,152,153]. In ERK5, this region undergoes intramolecular autophosphorylation at multiple residues under stress conditions that enhance the transcriptional activation of ERK5-dependent genes [154]. Additionally, the C-terminus of ERK5 also contains a nuclear localization signal (NLS), suggesting that autophosphorylation of this region may link ERK5 nuclear translocation and its transactivation function [155]. In an artificial system, when Slt2 is fused to the Gal4 DBD (DNA binding domain), it can activate *GAL1*-LacZ transcription in response to cell wall stress [156]. When Slt2 is artificially targeted to the promoter of CWI-dependent genes by the fusion of the MAPK to the Rlm1 DNA binding domain, it can bind to promoters and coding regions to induce certain levels of *MLP1* gene expression in the absence of Rlm1. Remarkably, the transcriptional induction of *MLP1* upon stress depends on MAPK phosphorylation, but not on its kinase activity. Thus, Slt2, by itself, can activate the transcription of CWI responsive genes independently of Rlm1, supporting a role for Slt2 in transcriptional activation as a structural component. Nevertheless, the presence of Rlm1 is still necessary for adequate gene induction [101]. 

Similarly, it has been shown that the phosphorylation of Slt2 but not its catalytic activity is required for flocculation, suggesting that Slt2 probably acts as a transactivator to induce transcription of *FLO* genes binding to their promoters via Rlm1, independently of its catalytic activity [157]. The binding of Rlm1 reduces the occupancy of the Tup1 repressor leading to the recruitment of TBP and Pol II at the promoters of *FLO* genes in flocculating cells. Antagonistic binding of Rlm1 and Tup1, and the presence of overlapping binding sites at the promoters of *FLO* genes suggest that the Rlm1 and Tup1 interplay is involved in the regulation of *FLO* gene expression and yeast flocculation [157] 

The ability of Slt2 to drive gene expression by itself is explained by the fact that Slt2 physically interacts with RNA Pol II upon cell wall stress. Slt2 is first recruited to promoters by Rlm1 to interact with RNA Pol II, to move from the transcription initiation complex to the transcription elongation complex at the coding region in a second step, independently of Rlm1 (Figure 3). This kinase recruitment mechanism differs from that described for Hog1, where Hog1 binding to coding regions is dependent on specific 3′ noncoding regions and, therefore, independent of promoter binding [158]. Besides, elongation of Rlm1-dependent genes is not controlled by Paf1C, although other elongation complexes such as THO and CCR4-NOT could be participating [101]. In accordance, it has also been suggested that ERK5 participates in elongation control, but the mechanism remains unclear [159]. The role of MAPK in transcriptional elongation, especially for Hog1, has been extensively studied. Hog1 is associated with the coding regions of osmostress genes and behaves as a transcriptional elongation factor by direct phosphorylation of the Spt4 elongation factor to regulate the activity of RNA Pol II [91,158,160]. Hog1 also interacts with the RSC chromatin remodeling complex to direct its association with the coding regions and modify nucleosome organization at this level [77]. 

Ubiquitination of H2B influences both transcriptional initiation and elongation [161,162] and involves changes in the organization and stability of chromatin to allow RNA Pol II to carry out transcription [163,164,165]. We have evidence that H2B ubiquitination by Rad6 (E2 Ubiquitin-conjugating enzyme) is necessary for H2B displacement at the coding region of CWI-responsive genes, in agreement with the participation of H2B ubiquitination in transcriptional elongation of genes regulated via the CWI pathway upon cell wall stress [166]. Moreover, Ubp8, a ubiquitin-specific protease component of the SAGA acetylation complex, is also recruited to coding regions to maintain appropriate levels of H2B ubiquitination (Figure 3). Thus, the main catalytic activities of the SAGA complex, acetylation and deubiquitination, are required for adequate expression of CWI-responsive genes.

### 3.2. Other CWI-Activating Conditions

In addition to cell wall damage conditions, other stimuli have also been found to trigger the activation of the CWI pathway [6]. The mechanisms of activation in these cases are much less understood. An interesting open question is how different stresses mount specific transcriptional responses by activation of the same MAPK. David Levin and co-workers have recently suggested that stress-specific MAPK outputs may be controlled, at least in part, by specific intracellular mechanisms of activation [167]. They uncovered the mechanisms by which DNA damage and arsenite stimulate the MAPKs Slt2 and Hog1, respectively. In both cases, the MAPK is activated through intracellular inputs (by the inhibition of phosphatases) that modulate their basal phosphorylation, rather than stimulating signaling through their corresponding protein kinase cascades [167,168,169]. In the case of arsenite, it inhibits Ptp2 and Ptp3, the tyrosine-specific phosphatases that maintain Hog1 in a low-activity state. Interestingly, the cellular response to arsenite fails to drive gene expression in support of glycerol production but contributes to arsenic-specific gene expression via the transcription factor Acr1 [167,170]. For genotoxic stress, the mechanism of activation of Slt2 includes induced ubiquitin-mediated proteolysis of the dual-specificity phosphatase Msg5, which maintains low levels of Slt2 activation in the absence of stress [169]. Similar to the case of arsenite, Slt2 activation by DNA damage does not drive the known cell wall integrity transcriptional program associated with cell wall stress, in agreement with the idea that specific MAPK outputs may be controlled by intracellular mechanisms. Additional observations suggest that protein kinase C (Pkc1) has a role in the DNA damage transcriptional response independent of its recognized function in the activation of Slt2 [171].

UPR^ER^, HOG, and CWI pathways are closely related and interact with each other in helping the cell to reduce cadmium toxicity [172]. Molecular mechanisms underlying transcriptional adaptive responses to cadmium and arsenate have also recently been reported [173]. The CWI pathway protects against both stresses through the upregulation of genes involved in cell wall biosynthesis and cell cycle control. The MAPK Slt2 exerts control of gene expression through distinct sets of CWI transcriptional regulators, including Rlm1 and SBF complex [173]. Thus, Rlm1, Swi4, and Swi6 transcription factors may coordinately modulate the expression of cell wall genes in response to cadmium-induced stress, whereas in response to arsenate-induced ER stress, Swi6 may play an important role in controlling the expression of cell cycle-regulating genes.

## 4. Conclusions and Future Perspectives

Understanding gene regulation in response to stress requires knowledge of the transcription mechanisms mediated by MAPKs. Regulatory mechanisms provide the cells with the necessary tools to respond efficiently to environmental changes. Tight regulation is important to ensure the correct temporal modulation of gene expression in response to stress. Gene expression under stress conditions is quite complex and requires not only the participation of MAPK and specific transcription factors, but also the coordinated action of different co-activators. Recruitment of co-activators and the transcription machinery to promoter regions is a key initial step in activating transcription. Although these regulatory mechanisms are not exclusive to each MAPK, different stress situations may mediate distinct molecular mechanisms at the transcriptional level with a diverse group of co-activators as protagonists to achieve coordinated and highly regulated responses. Even under the same type of stress, transcriptional mechanisms may vary depending on the architecture of the gene target.

In this review, we focus on the transcriptional mechanisms elicited by the MAPK Slt2 to activate gene expression under cell wall stress, highlighting the role of chromatin as an extra level of gene regulation. Nucleosome remodeling is crucial for inducible gene expression as it facilitates transcription activation. Upon cell wall stress, Slt2 phosphorylates and activates the main transcription factor Rlm1 to recruit both to the promoters of CWI-responsive genes in complex with SWI/SNF [83,101]. SWI/SNF activity is necessary to evict nucleosomes positioned at this region and permit pre-initiation complex (PIC) assembly. Nucleosome reorganization is also mediated by histone acetylation by the SAGA complex [82]. The role of the MAPK Slt2 in gene expression is not restricted to transcription initiation and PIC formation but extends to transcriptional elongation. Thus, Slt2 is also recruited to the coding regions of CWI-regulated genes, facilitating the progression of RNA Pol II along the ORF during transcription elongation [101]. In addition to the primary CWI transcriptional response, which is mediated by Rlm1, SBF (Swi4/Swi6) regulates the expression of a small group of genes, via a non-catalytic mechanism. In this case, Slt2 blocks premature transcription termination of cell wall stress genes, like *FKS2* regulated by SBF, via a mechanism that requires activation of Slt2 but not its catalytic activity [99].

Despite recent advances in molecular mechanisms controlling gene expression via the yeast CWI pathway, some questions remain unanswered. Studies have focused on the recruitment of transcription and chromatin regulatory complexes to chromatin. Still, little is known about how cells coordinate and discriminate activities and crosstalk between many gene regulatory factors in response to external cues. Combining genome-wide and context-specific approaches with chromosome conformation capture techniques will allow us to further understand the molecular mechanism involved in the genome organization of stress-responsive genes. Particularly, it will be interesting to see how each specific cell in a population responds to cell wall stress signals. Single-cell sequencing methods will allow monitoring the dynamics of mRNA production in single live cells and help elucidate global transcriptional responses and regulation mechanisms at the single-cell level. These mechanisms are beginning to be clarified in response to other stresses and their corresponding signaling pathways, like the HOG and UPR^ER^ signaling pathways. They suggest a particular interplay between MAPK signaling, chromatin, and transcription factors [174], and the possibility of effecting stress responses in different ways within distinct cells of an apparently homogeneous cell population [175,176]. A better understanding of cell wall stress responses at transcriptional and post-transcriptional stages will require additional stress-regulatory events, such as mRNA processing, transport of mRNAs from the nucleus to the cytoplasm, and regulation of mRNA stability, to be uncovered. Interestingly, cell wall stress induces the formation of P-bodies, and mRNAs, whose expression is regulated via the CWI pathway, localize to these structures [177].

MAPK pathways are essential for controlling cell physiology in all eukaryotes, and many of the signaling mechanisms mediated by MAPKs in yeasts are conserved in humans. A detailed characterization of the mechanisms required for regulating MAPK-mediated signaling and gene expression would allow the identification of novel targets for possible therapeutic intervention. ERK5, the human ortholog of Slt2, phosphorylates and activates several transcription factors, such as c-Myc, Sap1a, CREB, and the MEF2 family members MEF2A, C, and D, playing a critical role in cardiovascular development and vascular integrity [178,179]. Moreover, abnormal activation of this pathway has also been involved in pathological conditions leading to cancer and tumor angiogenesis [179,180,181]. Thus, the advances achieved in the characterization of the yeast MAPK Slt2 could be highly relevant, as many of its functions could be conserved. 

Treatments with cell wall perturbing agents, including antifungal inhibitors of β-1,3-glucan synthesis, like echinocandins, elicit rescue mechanisms in the budding yeast, particularly compensatory chitin synthesis, to maintain cellular integrity [5,102]. These adaptive responses, which are well conserved in pathogenic fungi including *Candida albicans* [182], *Aspergillus fumigatus* [183], *Candida auris* [184], and *Cryptococcus neoformans* [185], may decrease the effectiveness of antifungal treatments targeting cell wall biogenesis. Therefore, a better understanding of the mechanisms governing cell wall adaptive responses, including effector proteins and regulatory circuits, could allow the identification of new cellular targets for potential antifungal drugs. These responses are mainly regulated through the CWI pathway and other protein kinase signaling pathways like the HOG, calcineurin, and PKA pathways [102,185,186,187]. Thus, combining echinocandins with molecules interfering with antifungal adaptation pathways can be envisioned as a good antifungal strategy [188]. Cercosporamide, which acts selectively on Pkc1 kinase [189] is additive with echinocandins. Puupehenone, a marine-sponge-derived sesquiterpene quinone, also synergizes with echinocandins by inhibiting the interaction between Hsp90 and its cochaperone Cdc37, blocking the induction of caspofungin-responding genes required for adaptation to cell wall stress through the CWI pathway [190]. An alternative strategy in the same line of action would be to block some of the enzymatic activities associated with cell wall remodeling in these adaptation responses, including those necessary for the synthesis of compensatory chitin and glucan and enzymes involved in glucan remodeling and glucan-chitin crosslinking [191,192]. These enzymes play an important role in the fungal cell wall remodeling necessary to counterbalance cell wall stress, and many of them are transcriptionally induced in cell wall stress adaptive responses [102]. Thus, inhibitors of the chitin synthase such as nikkomycins, and inhibitors of the Gas/Phr β-1,3 glucanosyl transferases or Crh transglycosylases could be potentially used for combination therapies targeting the synthesis of β-1,3-glucan and blocking adaptive/compensatory cell wall remodeling mechanisms. Given the importance of stress adaptive response in the development of echinocandin resistance, these new combination therapies will likely be more effective in combating fungal pathogens.

## Data Availability

Not applicable.
